# Supplementary material for: A Revised Perspective on the Evolution of Troponin I and Troponin T Gene Families in Vertebrates
Source: Genome Biol Evol. 2022 Dec 15;15(1):evac173. doi: 10.1093/gbe/evac173 (PMC9825255; doi:10.1093/gbe/evac173)
Supplement: evac173_Supplementary_Data [file evac173_supplementary_data.zip › 20220417 Supp Materials Uncropped Blots.pdf]

Supplementary Materials

Uncropped original blots

Chemiluminescence detection

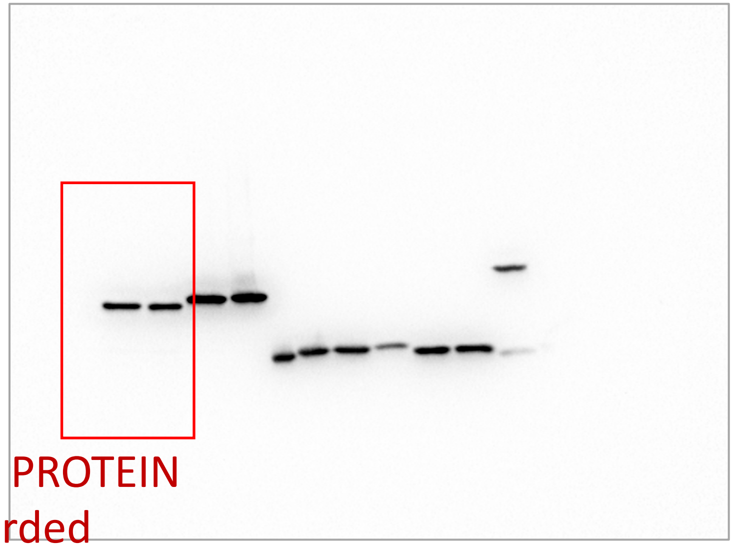

CATSHARK PROTEIN
OLD- discarded

Colorimetric image to show ladder, merged with chemiluminescent detection

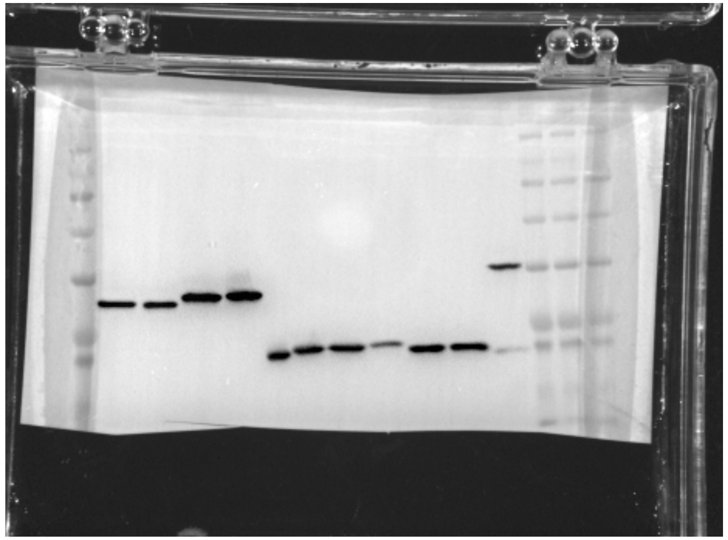

Lane number (left to right)	Lane ID	Notes
1	blank	
2	Ladder	
3	Catshark 3 (old)	Discarded and re-run because catshark protein was several years old and didn't show second smaller isoform and also showed reduced phosphorylation levels- sharks were re-run with freshly obtained samples
4	Catshark 1 (old)	
5	Greenland shark 1	
6	Greenland shark 2	
7	Bichir (2 ventricles pooled)	Fig 6B right panel
8	Sterlet 1	
9	Sterlet 2	
10	Gar (2 ventricles pooled)	
11	Eel 1	
12	Eel 2	
13	Lungfish 1	
14	Ladder	
15	Ladder	
16	Ladder	
17	blank	

Used in Figures:	6B
Gel system:	Invitrogen XCell SureLock Mini-Cell
Loading of lanes:	15 µg protein/lane<br><br>5µl BLUeye Prestained Protein Ladder (Sigma-Aldrich, 94964)
Gel:	SuperSep™ Ace 12.5% 17 Well pre-cast polyacrylamide gel
Transfer:	XCell II blot module (ThermoFisher)
Primary antibody	troponin I (C-4) SC-133117 1:1000 dilution
Secondary Antibody	m-IgGκ BP-HRP<br>SC-516102<br>1:5000 dilution
Chemiluminescent substrate	Bio-Rad Clarity Western ECL Substrate, 500 ml #1705061
Exposure time	4 s

Chemiluminescence detection

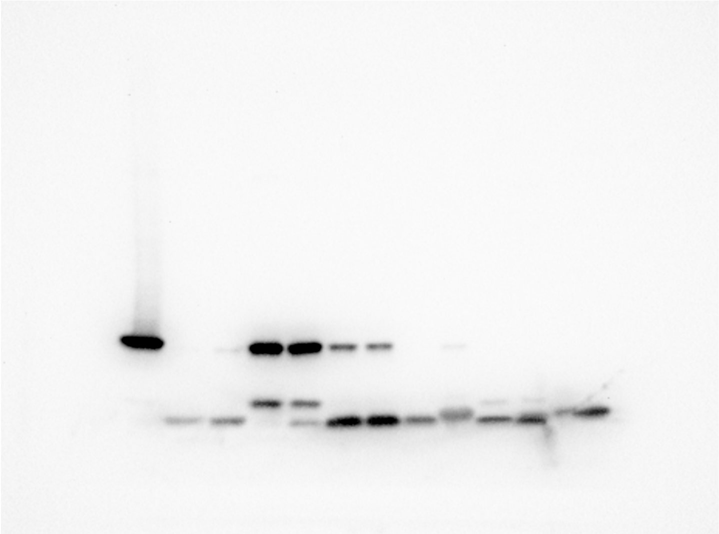

Colorimetric image to show ladder, merged with chemiluminescent detection

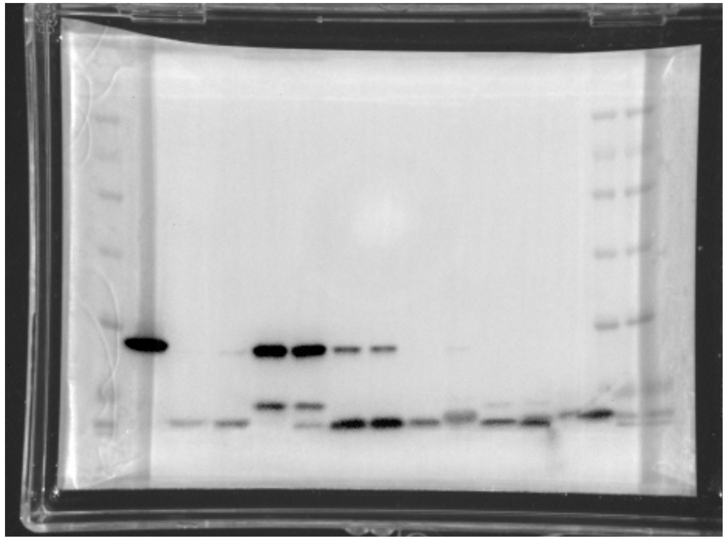

Lane number (left to right)	Lane ID
1	Ladder
2	Greenland shark 1 ventricle
3	Catshark A skeletal muscle
4	Catshark B skeletal muscle
5	Gs 1 red
6	Gs 2 red
7	Gs 1 white sk
8	Gs 2 white
9	Bichir sk musc
10	Sterlet sk musc
11	Gar skel musc
12	Eel skel musc
13	Lungfish skel musc
14	Ladder
15	Ladder

Used in Figures:	6D
Gel system:	Invitrogen XCell SureLock Mini-Cell
Loading of lanes:	15 µg protein/lane<br><br>5µl BLUeye Prestained Protein Ladder (Sigma-Aldrich, 94964)
Gel:	ThermoFisher Novex™ WedgeWell™ 10%, Tris-Glycine, 1.0 mm, Mini Protein Gel, 15-well
Transfer:	XCell II blot module (ThermoFisher)
Primary antibody	troponin I (C-4) SC-133117 1:1000 dilution
Secondary Antibody	m-IgGκ BP-HRP SC-516102 1:5000 dilution
Chemiluminescent substrate	Immobilon Classico Western HRP substrate Merck WBLUC0500
Exposure time	27 s

Chemiluminescence detection

Colorimetric image to show ladder merged with chemiluminescent detection

PKA

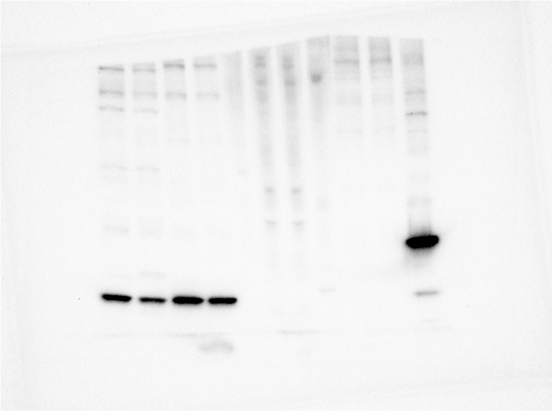

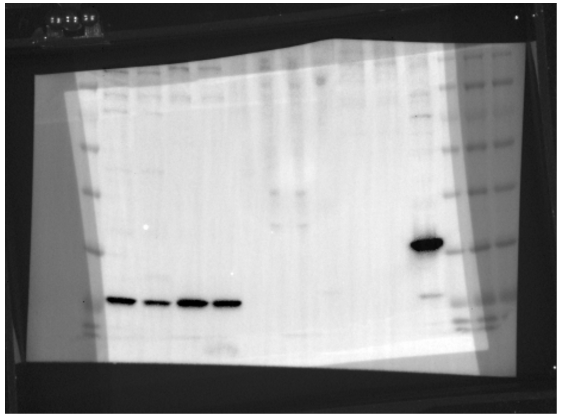

TnI

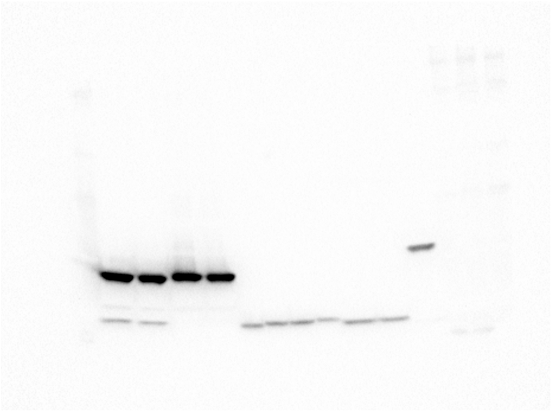

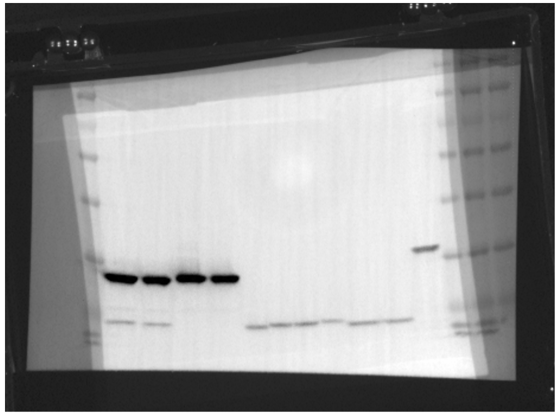

Lane number (left to right)	Lane ID
1	Ladder
2	Catshark A (new)
3	Catshark B (new)
4	Greenland shark 1
5	Greenland shark 2
6	Bichir (2 ventricles pooled)
7	Sterlet 1
8	Sterlet 2
9	Gar (2 ventricles pooled)
10	Eel 1
11	Eel 2
12	Lungfish M1
13	Ladder
14	Ladder
15	Ladder

Used in Figures:	7	7
Gel system:	Invitrogen XCell SureLock Mini-Cell	
Loading of lanes:	15 µg protein/lane<br><br>5µl BLUeye Prestained Protein Ladder (Sigma-Aldrich, 94964)	
Gel:	ThermoFisher Novex™ WedgeWell™ 10%, Tris-Glycine, 1.0 mm, Mini Protein Gel, 15-well	
Transfer:	XCell II blot module (ThermoFisher)	
Primary antibody	Cell Signaling Phospho-PKA Substrate (RRXS*/T*) (100G7E) Rabbit mAb #9624 1:1000 dilution	troponin I (C-4) SC-133117 1:1000 dilution
Secondary Antibody	Anti-rabbit IgG, HRP-linked Antibody #7074 1:3000 dilution	m-IgGκ BP-HRP SC-516102 1:5000 dilution
Chemiluminescent substrate	Bio-Rad Clarity Western ECL Substrate, 500 ml #1705061	Bio-Rad Clarity Western ECL Substrate, 500 ml #1705061
Exposure time	19.7 s	7 s

Chemiluminescence detection

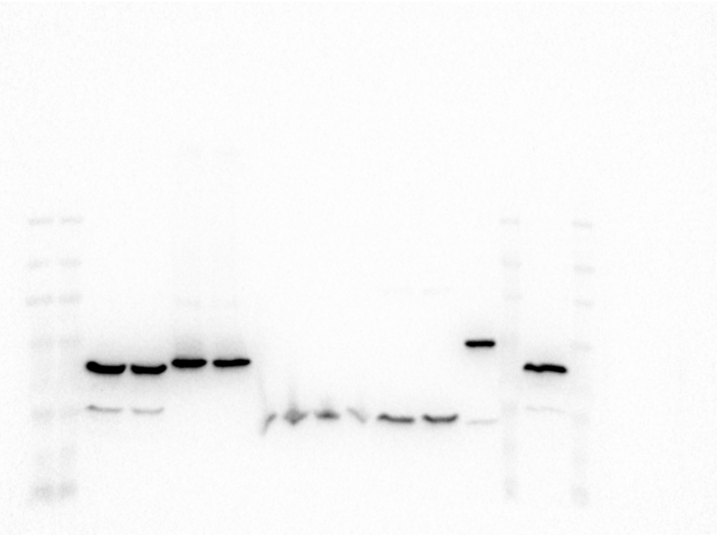

Colorimetric image to show ladder, merged with chemiluminescent detection

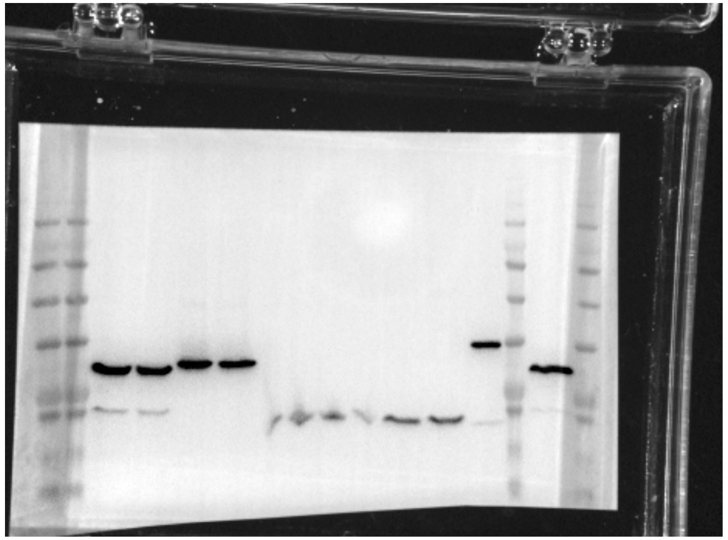

Lane number (left to right)	Lane ID	
1	Ladder	
2	Ladder	
3	Catshark A	Sharks that had been re-run with fresh samples (see notes on Slide 3)
4	Catshark B	
5	Greenland shark 1	
6	Greenland shark 2	
7	Bichir (2 ventricles pooled)	Discarded as lanes had compressed and clearer results in previous run (see first blot)
8	Sterlet 1	
9	Sterlet 2	
10	Gar (2 ventricles pooled)	
11	Eel 1	
12	Eel 2	
13	Lungfish M1	
14	Ladder	
15	Catshark atrium (trial)	
16	Ladder	
17	blank	

Used in Figures:	6B left panel
Gel system:	Invitrogen XCell SureLock Mini-Cell
Loading of lanes:	15 µg protein/lane<br><br>5µl BLUeye Prestained Protein Ladder (Sigma-Aldrich, 94964)
Gel:	SuperSep™ Ace 12.5% 17 Well pre-cast polyacrylamide gel
Transfer:	XCell II blot module (ThermoFisher)
Primary antibody	troponin I (C-4) SC-133117 1:1000 dilution
Secondary Antibody	m-IgGκ BP-HRP SC-516102 1:5000 dilution
Chemiluminescent substrate	Immobilon Classico Western HRP substrate Merck WBLUC0500
Exposure time	7 s
