# Supplementary material for: A Revised Perspective on the Evolution of Troponin I and Troponin T Gene Families in Vertebrates
Source: Genome Biol Evol. 2022 Dec 15;15(1):evac173. doi: 10.1093/gbe/evac173 (PMC9825255; doi:10.1093/gbe/evac173)
Supplement: evac173_Supplementary_Data [file evac173_supplementary_data.zip › R1 Supplementary Figures S1-S6 NEW.pdf]

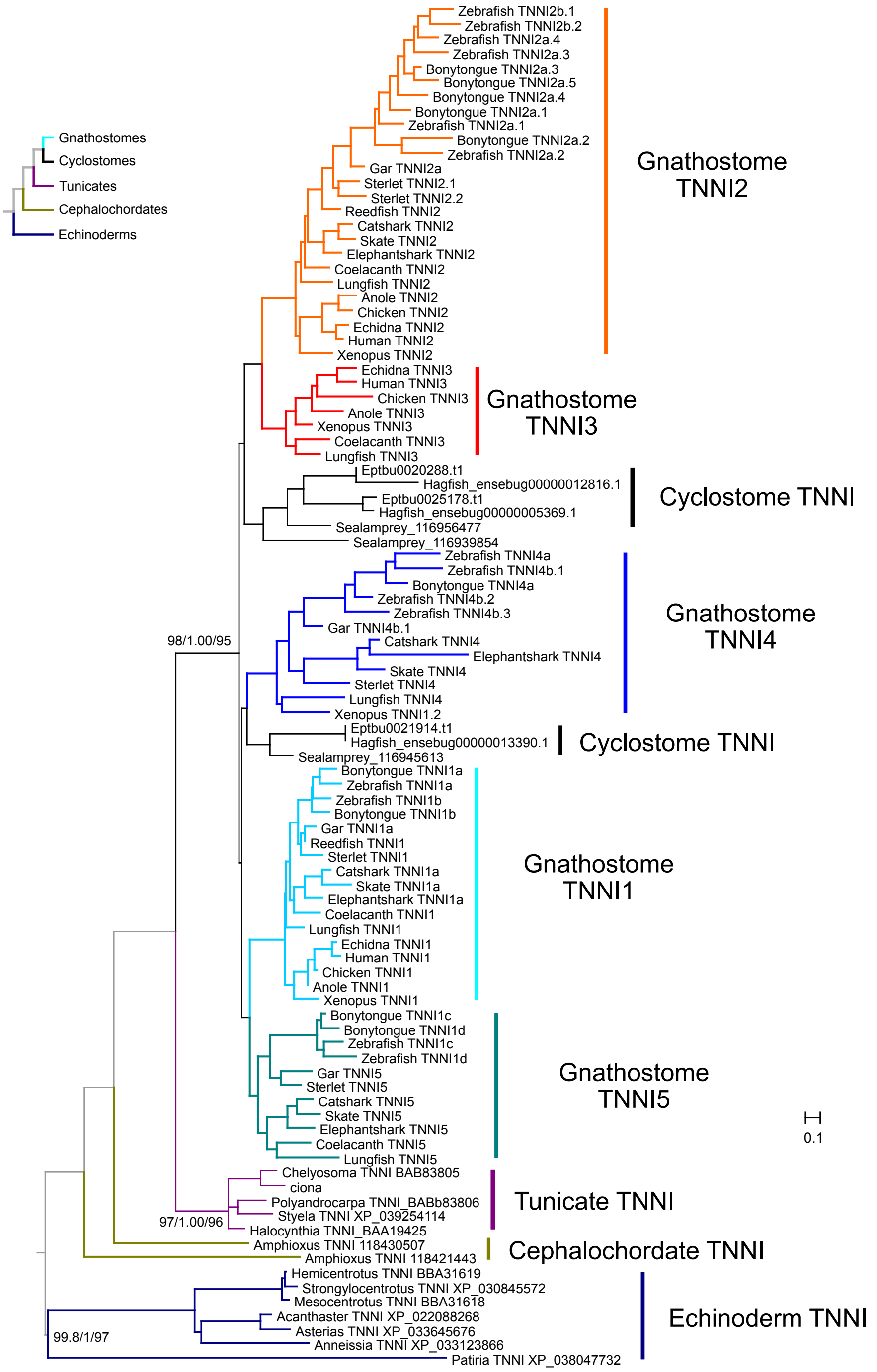

Supplementary Figure S1. Maximum likelihood phylogenetic tree showing evolutionary relationships between troponin I (*TNNI*) sequences from vertebrates and a range of invertebrate deuterostome outgroups, including tunicates, cephalochordates and echinoderms. Support for the relevant nodes is shown as SH-aLRT support (%) (Guindon et al. 2010) / aBayes support (Anisimova et al. 2011) / ultrafast bootstrap support (%) (Hoang et al. 2018).

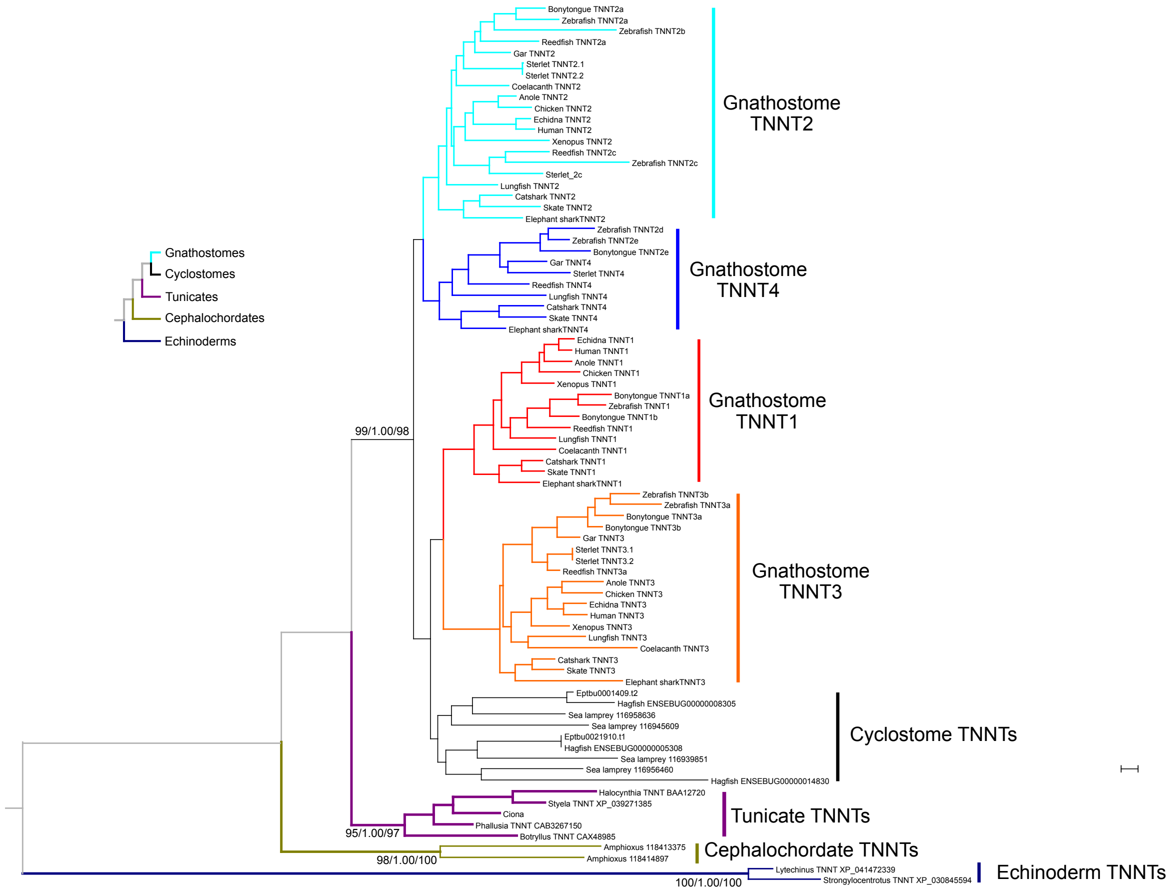

Supplementary Figure S2. Maximum likelihood phylogenetic tree showing evolutionary relationships between troponin T (*TNNT*) sequences from vertebrates and a range of invertebrate deuterostome outgroups, including tunicates, cephalochordates and echinoderms. Support for the relevant nodes is shown as SH-aLRT support (%) / aBayes support / ultrafast bootstrap support (%).

>catsharkTNNI5
MSDQEEYEEEVVEEMEETEEVIESEPEPPKPAPPPKAAPAPRGPGAQKETIAKKSC
KISASRKLHLKILMLGKAKEDLEKETVDRNEEKEKFLAERVPPLNFTGLSLTDLQNLC
KELHQRIEIVDEERYDFEFKAGKNNYEIHDLSLKILDLRGKFKRPTLRRVRVSADAML
RALLGSKHKVSMDLRANLKSVKKDDTEKERNVEVSDWRKNVEAKSGMEGRKKMF
DAANQ

cu|M00501|csSSX, 26,514.1 Da
csSSX (William Joyce (Shiels))
7 exclusive unique peptides, 7 exclusive unique spectra, 18 total spectra, 65/231 amino acids (28% coverage)

MSDQEEYEEE VVEEMEETEE VIESEPEPPK PAPPPKAAPA PRGPGAQKET IAKKSCKISA
SRKLHLKILM LGKAKEDLEK ETVDRNEEKE KFLAERVPPL NFTGLSLTDL QNLCKELHQR
IEIVDEERYD FEFKAGKNNY EIHDLSLKIL DLRGKFKRPT LRRVRVSADA MLRALLGSKH
KVSMDLRANL KSVKKDDTEK ERNVEVSDWR KNVEAKSGME GRKKMFDAAN Q

>greenlandsharkTNNI5
MSDQEEQYEEEVIDETEETKEVIEPEPEPPKPAPPKQVAPPPRPQVAPHEINAKKS
CKISASRKLHLKILMLGKAKDDLEKEIVDRNEEKEKFLAERVPPLNLSGLSLTDLQNL
CIELHQKIEIVDEERYDFEFKAGKNSYEIHDLSLKILDLRGKFKRPTLRRVRVSADAM
LRALLGSKHKVSMDLRANLKSVKKDDTEKERNVEVSDWRKNVEAKSGMEGRKKM
FDAASAQ

cu|M00484|GSSSX_GLAND, 26,734.5 Da
greenlandsharkSSX (William Joyce (Shiels))
45 exclusive unique peptides, 46 exclusive unique spectra, 105 total spectra, 151/233 amino acids (65% coverage)

MSDQEEQYEE EVIDETEETK EVIEPEPEPP KPAPPKQVAP PPRPQVAPHE INAKKSCKIS
ASRKLHLKIL MLGKAKDDLE KEIVDRNEEK EKFLAERVPP LNLSGLSLTD LQNLCIELHQ
KIEIVDEERY DFEFKAGKNS YEIHDLSLKI LDLRGKFKRP TLRRVRVSAD AMLRALLGSK
HKVSMDLRAN LKSVKKDDTE KERNVEVSDW RKNVEAKSGM EGRKKMFDAA SAQ

>africanlungfishTNNI3
MADEEEVTQYEEEEEYADEEEAEEEEEVKEFEPAKPATPPPPPAAPAPLTRRPSSL
NYRSLVAQPQVKRKSKITASRKLQLKSLMLQIAKQELEREAEERAEEKERYLTQRC
EPLQLSGFSLVELQDLCKQLHATVDVADEERYDLEAKVSKNVQEIEDLNQKIFDLRG
KFKLPQLRRVRMSADAMLRALLGSKHKVCMDLRANLKQVKKDDVEKEIREVGDWR
KNIDAMAGMEGRKKKFEFSLSGQA*

cu|M00482|ALFC_LFISH, 28,632.0 Da
africanlungfishC (William Joyce (Shiels))
97 exclusive unique peptides, 121 exclusive unique spectra, 166 total spectra, 205/248 amino acids (83% coverage)

MADEEEVTQY EEEEEYADEE EAEEEEEVKE FEPAKPATPP PPPAAPAPLT RRPSSLNYRS
LVAQPQVKRK SKITASRKLQ LKSLMLQIAK QELEREAEER AEEKERYLTQ RCEPLQLSGF
SLVELQDLCK QLHATVDVAD EERYDLEAKV SKNVQEIEDL NQKIFDLRGK FKLPQLRRVR
MSADAMLRAL LGSKHKVCMD LRANLKQVKK DDVEKEIREV GDWRKNIDAM AGMEGRKKKF
EFSLSGQA

Supplementary Figure S3. Mass spectrometry results showing transcriptomic-predicted *TNNI5* and *TNNI3* sequences from sharks and lungfish, respectively, annotated to show peptide matches (highlighted in yellow) and corresponding protein identification coverage.

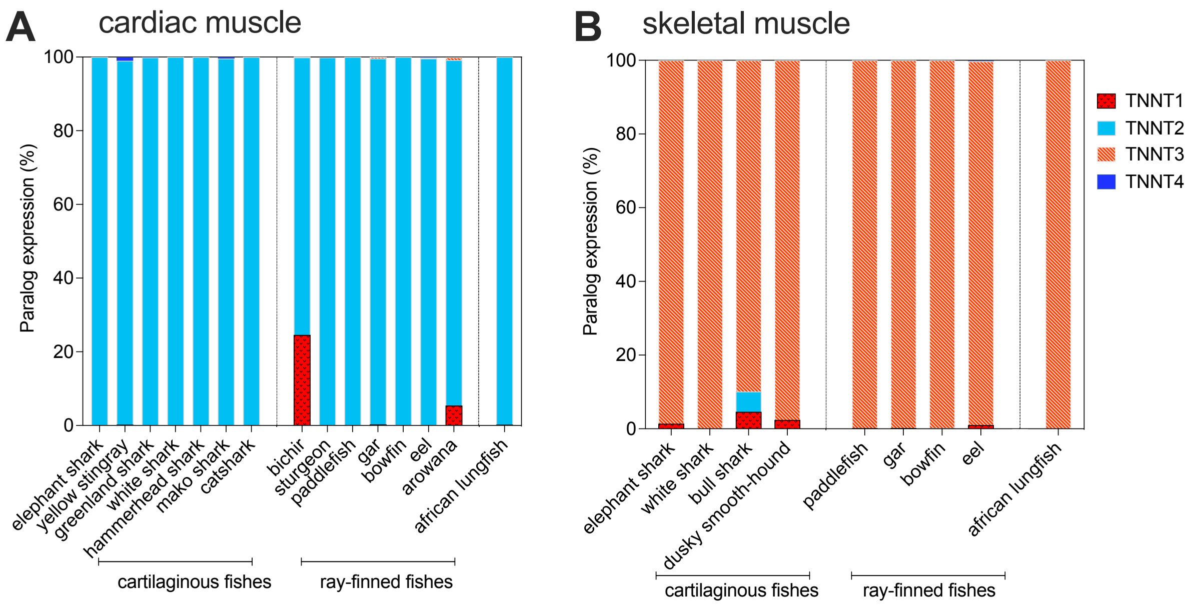

Supplementary Figure S4. *TNNT* gene expression in cartilaginous fishes, ray-finned fishes and lungfish from transcriptomic analysis, including cardiac (A) and skeletal muscle (B). Transcript identity was assigned using blastp queried against *TNNT* protein sequences for catshark.

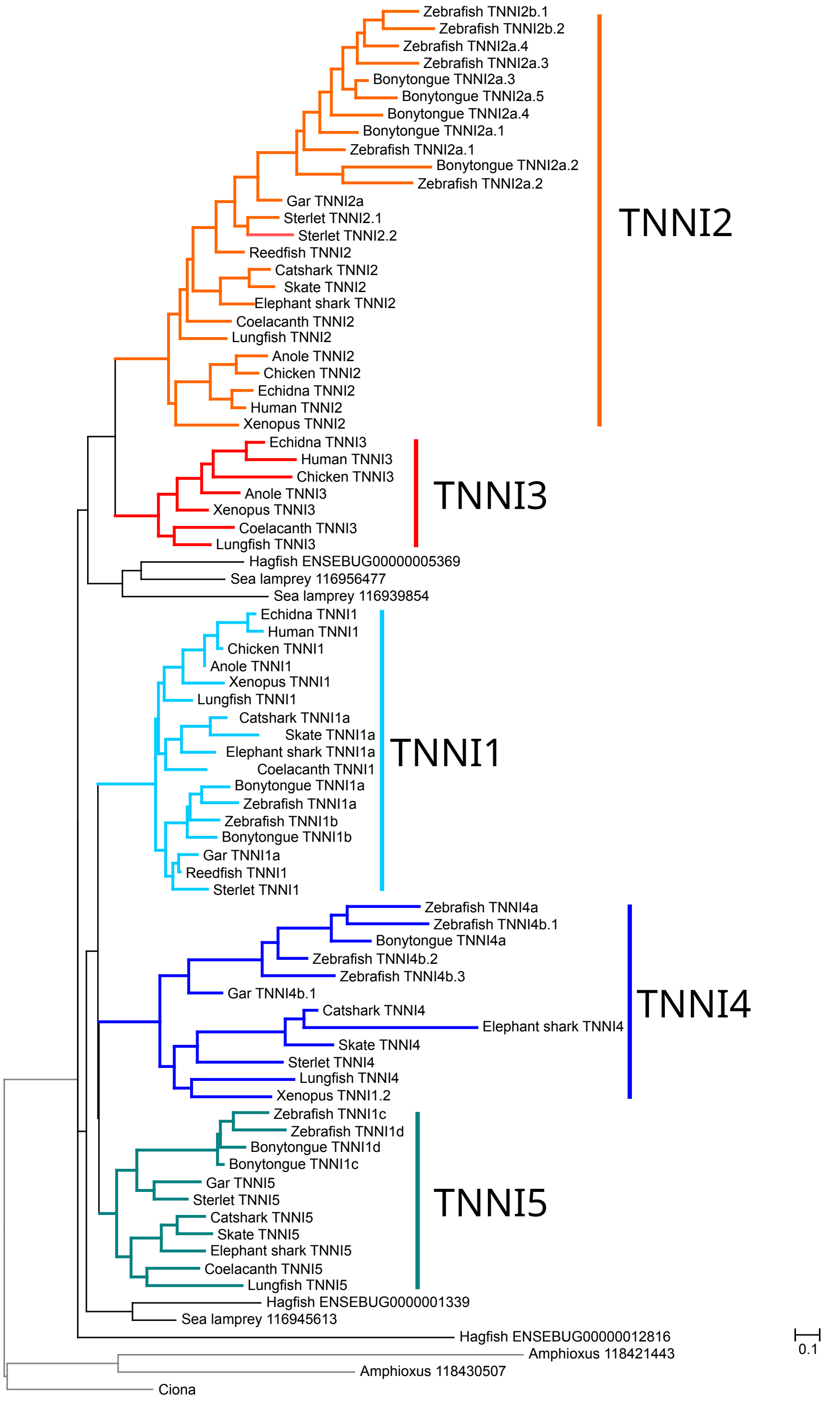

Supplementary Figure S5. *TNNI* phylogenetic tree constrained to show *TNNI4*-*TNNI5* sister relationship. The tree was not significantly different from the maximum likelihood tree (Fig. 1) which showed *TNNI1*-*TNNI5* as sister (Supplementary Table 2).

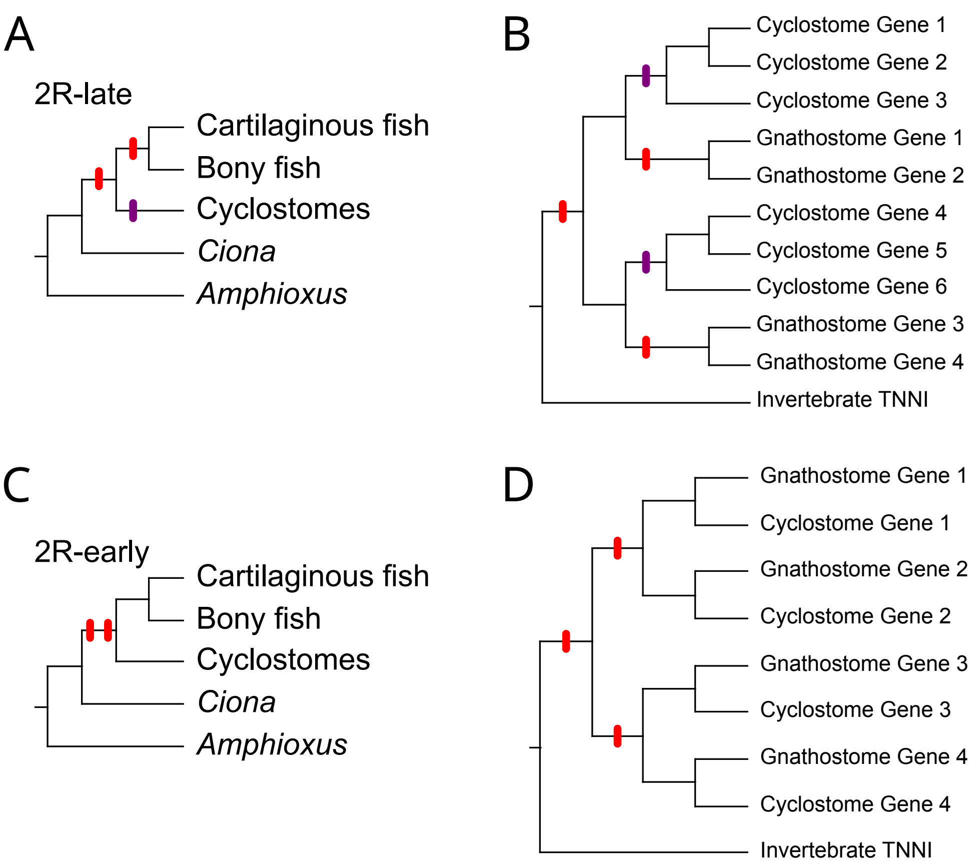

Supplementary Figure S6. Alternative hypotheses regarding the timing of the two rounds of whole genome duplication early in vertebrate evolution and their predictions regarding phylogenetic relationships the descending paralogs. There is consensus that gnathostomes underwent two rounds of WGD (Meyer & Schartl 1999; McLysaght et al. 2002; Dehal & Boore 2005), 1R and 2R (shown as red bars), and that 1R predates the split of cyclostomes and gnathostomes. However, the placement of 2R on the vertebrate tree is debated (Kuraku et al. 2009). Recent studies place 2R in the last common ancestor of gnathostomes (Simakov et al. 2020; Nakatani et al. 2021), *i.e.* '2R-late', and suggest that cyclostomes underwent an independent polyploidization early in their evolution (Mehta et al. 2013; Nakatani et al. 2021). By contrast, some authors place 2R in the common ancestor of cyclostomes and gnathostomes (Sacerdot et al. 2018), i.e. '2R-early'. Panels A and C show the placement of these polyploidizations on the vertebrate tree. Panels B and D show the competing phylogenetic predictions.

References

Anisimova M, Gil M, Dufayard J-F, Dessimoz C, Gascuel O. 2011. Survey of Branch Support Methods Demonstrates Accuracy, Power, and Robustness of Fast Likelihood-based Approximation Schemes. Systematic Biology. 60:685–699. doi: 10.1093/sysbio/syr041.

Guindon S et al. 2010. New Algorithms and Methods to Estimate Maximum-Likelihood Phylogenies: Assessing the Performance of PhyML 3.0. Systematic Biology. 59:307–321. doi: 10.1093/sysbio/syq010.

Hoang DT, Chernomor O, von Haeseler A, Minh BQ, Vinh LS. 2018. UFBoot2: Improving the Ultrafast Bootstrap Approximation. Molecular Biology and Evolution. 35:518–522. doi: 10.1093/molbev/msx281.
