# Supplementary material for: A Revised Perspective on the Evolution of Troponin I and Troponin T Gene Families in Vertebrates
Source: Genome Biol Evol. 2022 Dec 15;15(1):evac173. doi: 10.1093/gbe/evac173 (PMC9825255; doi:10.1093/gbe/evac173)
Supplement: evac173_Supplementary_Data [file evac173_supplementary_data.zip › Supplementary Tables 1-3.pdf]

Supplementary Table 1. Proposed changes in nomenclature for previously mis-annotated genes. Revised proposed names are intended to reflect the evolutionary history and phylogenetic affiliations within newly defined *TNNI* and *TNNT* groups.

**Lineage**	**Current name**	**Proposed revision**
Amphibians	*tnni1.2*	*tnni4*
Teleost fishes	*tnni1c/ tnni1d*	*tnni5a/ tnni5b*
Teleost fishes	*tnnt2d/ tnni2e*	*tnnt4a/ tnni4b*

Supplementary Table 2. Statistical comparisons of constrained trees.

TNNI

Tree	logL	deltaL	bp-RELL	p-KH	p-SH	c-ELW	p-AU
1	-14437.73	0	0.553	0.776	1	0.534	0.768
2	-14442.38	4.7	0.301	0.32	0.356	0.297	0.336
3	-14439.80	2.1	0.146	0.224	0.551	0.169	0.233

Tree 1: Unconstrained maximum likelihood tree (figure

Tree 2: Gnathostome TNNI4 constrained as sister to Gnathostome TNNI5

Tree 3: Constrained to match the 2R-late hypothesis.

TNNT

Tree	logL	deltaL	bp-RELL	p-KH	p-SH	c-ELW	p-AU
1	-19442.77	0	0.896	0.896	1	0.895	0.904
2	-19467.96	25.191	0.104	0.104	0.104	0.105	0.0958

Tree 1: Unconstrained maximum likelihood tree (figure

Tree 2: Constrained to match the 2R-late hypothesis.

deltaL : logL difference from the maximal logl in the set.

bp-RELL : bootstrap proportion using RELL method (Kishino et al. 1990).

p-KH : p-value of one sided Kishino-Hasegawa test (1989).

p-SH : p-value of Shimodaira-Hasegawa test (2000).

c-ELW : Expected Likelihood Weight (Strimmer & Rambaut 2002).

p-AU : p-value of approximately unbiased (AU) test (Shimodaira, 2002).

Supplementary Table 3

Tracing of human, sea lamprey and amphioxus genes to the proto-gnathostome, proto-cyclostome and proto-vertebrate chromosomes defined by Nakatani et al. (2021)

Human

Gene name	NCBI Gene ID	NCBI Protein ID	Ensembl Gene ID	Ensembl Protein ID	Genomic segment	Chromosome	Proto-gnathostome Chromosome	Proto-vertebrate Chromosome
TNNI1	7135	NP_003272	ENSG00000159173	ENSP00000354488	Human_10	1	24	Pv11
TNNI2	7136	NP_003273	ENSG00000130598	ENSP00000371331	Human_81	11	25	Pv11
TNNI3	7137	NP_000354	ENSG00000129991	ENSP00000341838	Human_130	19	27	Pv11

Sea lamprey

Gene Name	NCBI Gene ID	kPetMar1 ID	kPetMar1 chr.	gPMar100 ID	gPMar100 scaffold	Lamprey chromosome fragment	proto cyclostome	proto vertebrate chromosome
TNNI	116939854	PMZ_0053578-RA	7	PMZ_0008467-RA	scaf_00014	Sea Lamprey_44	Pcc11A	Pv11
	116939854	PMZ_0053578-RA		(PMZ_0047782-RA)				
TNNI	116956477	PMZ_0061710-RA	65	PMZ_0033355-RA	scaf_00064	Sea Lamprey_106	Pcc11E	Pv11
TNNI	116945613	PMZ_0058172-RA	24	PMZ_0040924-RA	scaf_00002	Sea Lamprey_15	Pcc11B	Pv11

Amphioxus

Gene	Gene ID	Protein ID Bfl_VNyyK (GCA_000003815.2)	Chromosome Bfl_VNyyK	Protein ID Version 2 (GCF_000003815)	scaffold version 2	proto vertebrate chromosome
TNNT	118413375	XP_035672621	4	missing in annotation	scaffold 230	Pv11
TNNT	118414897	XP_035675082	4	XP_002590197	scaffold 230	Pv11
